# FASN inhibition targets multiple drivers of NASH by reducing steatosis, inflammation and fibrosis in preclinical models

**DOI:** 10.1038/s41598-022-19459-z

**Published:** 2022-09-19

**Authors:** Marie O’Farrell, Greg Duke, Richard Crowley, Douglas Buckley, Eduardo B. Martins, Dipankar Bhattacharya, Scott L. Friedman, George Kemble

**Affiliations:** 1 Sagimet Biosciences Inc., 155 Bovet Rd, San Mateo, CA 94402 USA; 2 Division of Liver Diseases, Icahn School of Medicine at Mount Sinai, New York, NY USA

**Keywords:** Mechanisms of disease, Endocrine system and metabolic diseases, Pharmacology, Gastroenterology

## Abstract

Fatty acid synthase (FASN) is an attractive therapeutic target in non-alcoholic steatohepatitis (NASH) because it drives de novo lipogenesis and mediates pro-inflammatory and fibrogenic signaling. We therefore tested pharmacological inhibition of FASN in human cell culture and in three diet induced mouse models of NASH. Three related FASN inhibitors were used; TVB-3664, TVB-3166 and clinical stage TVB-2640 (denifanstat). In human primary liver microtissues, FASN inhibiton (FASNi) decreased triglyceride (TG) content, consistent with direct anti-steatotic activity. In human hepatic stellate cells, FASNi reduced markers of fibrosis including collagen1α (COL1α1) and α-smooth muscle actin (αSMA). In CD4+ T cells exposed to NASH-related cytokines, FASNi decreased production of Th17 cells, and reduced IL-1β release in LPS-stimulated PBMCs. In mice with diet induced NASH l, FASNi prevented development of hepatic steatosis and fibrosis, and reduced circulating IL-1β. In mice with established diet-induced NASH, FASNi reduced NAFLD activity score, fibrosis score, ALT and TG levels. In the CCl4-induced FAT-NASH mouse model, FASN inhibition decreased hepatic fibrosis and fibrosis markers, and development of hepatocellular carcinoma (HCC) tumors by 85%. These results demonstrate that FASN inhibition attenuates inflammatory and fibrotic drivers of NASH by direct inhibition of immune and stellate cells, beyond decreasing fat accumulation in hepatocytes. FASN inhibition therefore provides an opportunity to target three key hallmarks of NASH.

## Introduction

Nonalcoholic fatty liver disease (NAFLD) is a global epidemic associated with obesity, type 2 diabetes (T2D), insulin resistance and metabolic syndrome, and is histologically associated with fat accumulation (steatosis) in the liver. Nonalcoholic steatohepatitis (NASH) is an aggressive, progressive form of NAFLD in which there is hepatocyte injury, infiltration by inflammatory cells and activation of fibrogenic stellate cells^1^. Activation of stellate cells leading to fibrosis also confers increased risk of progression to cirrhosis and hepatocellular carcinoma (HCC). Hepatic fibrosis is the sole histologic predictor of liver related outcomes in patients with NASH^2–4^.

Fatty acid synthase (FASN) is an enzyme in the de novo lipogenesis (DNL) pathway that converts the metabolites of dietary sugars, acetyl-coenzyme A (CoA) and malonyl-CoA, into palmitate, a saturated fatty acid at the final committed step of the DNL pathway. Hepatic DNL is increased in patients with metabolic syndrome and NAFLD compared to healthy individuals^5,6^, associated with increased intake of dietary sugars. FASN gene expression is elevated in liver biopsies from NAFLD patients^7^.

Palmitate generated by FASN has several possible fates in the liver of patients with NAFLD. First, in hepatocytes, it is a building block for synthesis of fatty acids and more complex lipids such as triglycerides that generate steatosis. Second, the DNL pathway is essential for human hepatic stellate cell (hHSC) activation and fibrogenesis, and its inhibition leads to HSC quiescence and decreased collagen production^8,9^. HSCs are rich in lipid droplets containing retinyl esters and are quiescent in normal liver. Liver injury provokes transdifferentiation of HSCs into myofibroblast-like cells which lose lipid and acquire a fibrogenic, contractile and proliferative phenotype^10^. The LX-2 cell line is an activated human HSC that harbors key features of activated HSCs^10,11^ and is a widely used platform for initial screening of candidate antifibrotic drugs^12^. Third, palmitate is a substrate for synthesis of pro-inflammatory and pro-fibrotic lipotoxins including ceramides, sphingomyelins and diacylglycerols (DAGs)^13^. Lipotoxicity is a major contributor to the pathogenic mechanisms driving the progression of NASH^14^. NASH patients have increased levels of ceramides that are associated with insulin resistance, inflammation, and production of reactive oxygen species^15–17^. Increased intrahepatic fat and the generation of lipotoxic metabolites promote the progression of NAFLD by damaging hepatocytes, stimulating inflammatory responses, and activating HSCs. Fourth, palmitate directly activates the Nod-like receptor (NLR) family pyrin domain containing 3 (NLRP3) inflammasome. In human macrophages palmitic acid treatment leads to pro-inflammatory cytokine production^18^. In rat HSCs, administration of palmitic acid activates the NLRP3 inflammasome via the TLR4 pathway and leads to activation, proliferation and expression of pro-fibrogenic genes such as αSMA, Col1α1, TIMP1 and TGF-beta^19^. Palmitate administration can directly cause liver injury and NASH in mouse models^20^. The DNL pathway is also important for development of pro-inflammatory T helper (Th17) cells^21,22^, which are increased in the liver of NASH patients compared to healthy liver and may contribute to stellate cell activation and liver damage. This may provide another mechanism for FASN inhibition to decrease liver inflammation.

Based on the disease-promoting properties of the DNL pathway and palmitate, FASN is an attractive drug target to attenuate hallmarks of NASH pathogenesis driven by hepatocytes, immune cells and HSCs. Early tool FASN inhibitors included natural products such as cerelulin and platensimycin, and a covalent inhibitor named C-75 which have been useful to explore the biology of FASN in a range of preclinical models, but have off-target activities and are not suitable for clinical development [review, ^23^]. We tested potent small molecule FASN inhibitors including TVB-2640 (denifanstat) in liver, immune and fibrogenic cells ex vivo, and in mouse models of NASH and liver fibrosis. The efficacy observed underscores the value of FASN inhibition in NASH, and provides insight into mechanism of action. These results support the rationale underlying clinical testing of denifanstat, a first in class FASN inhibitor currently in Phase 2 for NASH.

## Results

### Description of FASN inhibitors

Three chemically related FASN inhibitors were used in these studies. They were developed at Sagimet Biosciences (formerly 3-V Biosciences) and have been previously described^24–26^. All three are highly potent and selective FASN inhibitors that target the β-ketoreductase domain. A summary of compound characteristics is provided in Table 1. FASN inhibitors were tested in different NASH models based on DMPK properties and development stage. Briefly, TVB-3664 has optimized mouse PK and potency, and was therefore used for the in vivo mouse NASH models^26^, and in the human hepatic stellate cell (HSC) and T cell studies. TVB-2640 (denifanstat) is in clinical development for NASH^24^ and was used in the primary human liver microtissue (LMT) study. The third FASN inhibitor, TVB-3166, is a well characterized first generation Sagimet FASNi^25,26^ and was used in the PBMC study.

**Table 1:** Characteristics of FASN inhibitors TVB-2640 (denifanstat), TVB-3664 and TVB-3166.

	TVB-2640 Denifanstat	TVB-3664	TVB-3166
Status	In clinical development	2nd generation FASN inhibitor for preclinical testing	1st generation FASN inhibitor for preclinical testing
In vitro FASN biochemical inhibition: human IC_50_ (µM)	0.044	0.026	0.049
In vitro palmitate inhibition: human cells IC_50_ (µM)	0.030 Hela 0.048 Huh7	0.018 Hela	0.070 Hela
In vitro palmitate inhibition—mouse cells IC_50_ (µM)	0.543 CT26	0.012 CT26 0.016 C57Bl6	0.86 CT26
Model used in the studies described	Human ex-vivo liver microtissue	Mouse in vivo models Human ex-vivo Th17 Human in vitro LX-2 stellate cell line Human ex-vivo primary hepatic stellate cells	Human PBMC
Mouse PK	Poor	Best	Good
Clinical development	Phase 2b NASH	No	No
Structure			
Reference	^24,36^	^26,35^	^25,26^

### FASNi treatment reduces cellular triglycerides in human liver microtissues

**Figure 1:**
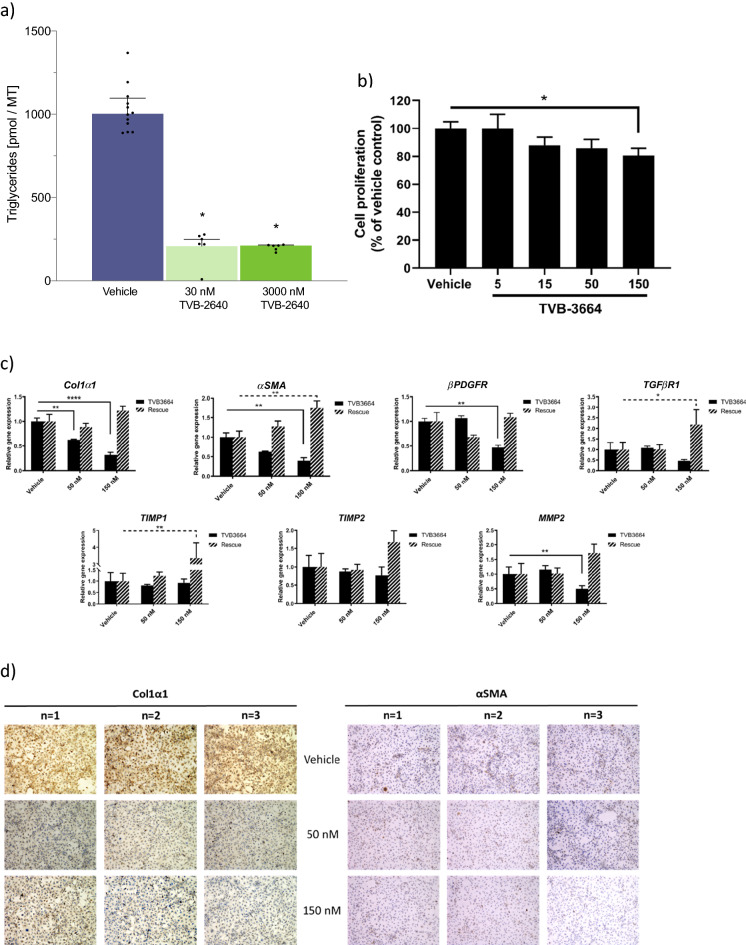

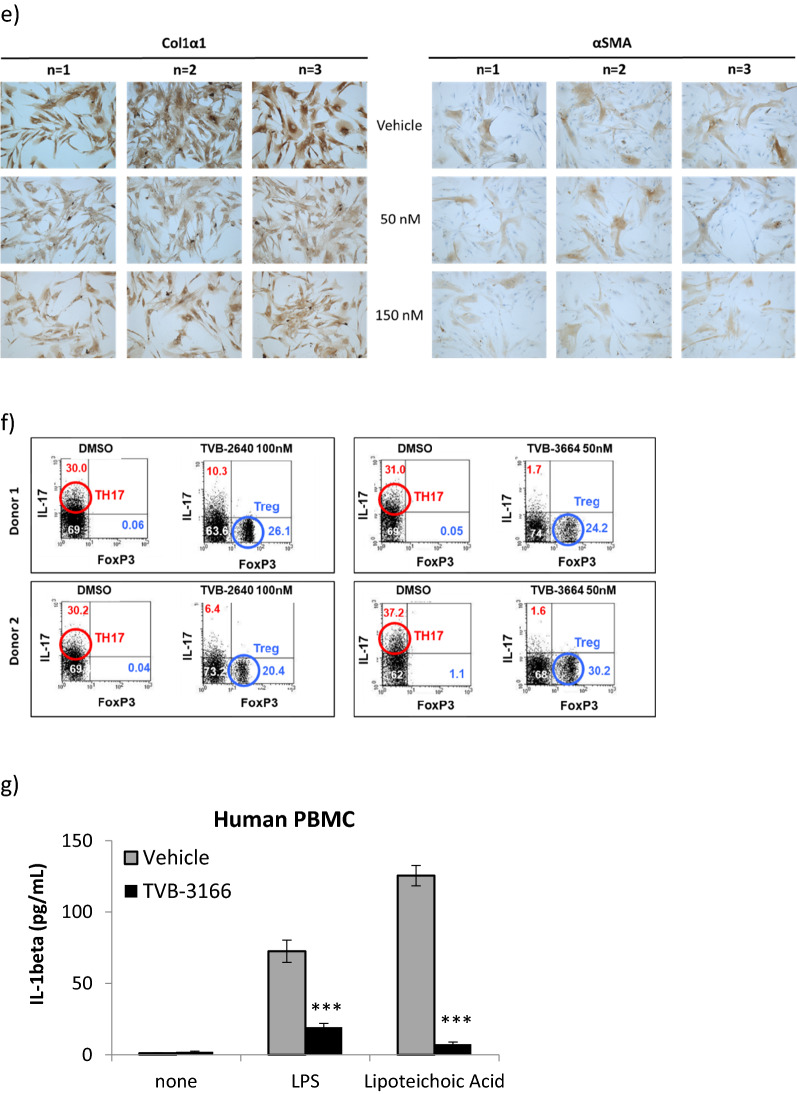
In human in vitro models, FASN inhibition directly reduces cellular triglyceride content, fibrogenic marker expression, pro-inflammatory cytokine production and Th17 cell development. (**a**) Effect of FASNi on TG content in Liver Micro Tissues (LMTs) were treated for 10 days with TVB-2640 in the presence of glucose, insulin and fatty acids, and assayed for TG levels. Mean ± SD of 12 replicates for vehicle and 6 for each TVB-2640 condition. *p < 0.05 by unpaired T test versus vehicle. (**b**) Effect of FASNi on cellular proliferation in LX-2 cells. *p < 0.05 by unpaired T test versus vehicle. (**c**) Effect of FASNi on expression of fibrogenic genes in LX-2 cells. *p < 0.01, **p < 0.05, ****p < 0.0001 by unpaired T test versus vehicle. Quantitative PCR analysis was performed after 48 h of treatment (solid bars) with TVB-3664 followed by washout to remove drug and a further 48 h incubation (striped bars). (**d**) Effect of FASNi on protein expression of Col1α1 and αSMA in LX-2 cells. Immunocytochemistry in cells exposed to 50 or 150 nM TVB-3664 for 48 h. Three independent experiments are indicated by n = 1, 2 or 3. (**e**) Effect of FASNi on protein expression of Col1α1 and αSMA in primary human hepatic stellate cells (hHSC) cells. Immunocytochemistry in cells exposed to 50 or 150 nM TVB-3664 for 48 h. Three independent experiments are indicated by n = 1, 2 or 3. (**f**) Effect of FASNi on Th17 cell development. Flow cytometry analysis of Th17 (IL-17 marker, red circle) and Treg cells (FoxP3 marker, blue circle) cells developed from human CD4+ cells. Two independent human donors are shown, cultured in the absence (DMSO control) or presence of FASNi. (**g**) Effect of FASNi on IL-1β levels in primary human PBMC. Analysis of supernatant after 48 h of treatment with LPS or lipotechoic in the absence or group presence of TVB-3166. Mean/SD. ***p < 0.001 by paired T test versus vehicle.

The in vitro effect of FASN inhibition on cellular triglycerides was tested in a primary human liver microtissue (LMT) model that contains hepatocytes as the majority cell type, in addition to hepatic endothelial cells, hepatic stellate cells (HSC) and Kupffer cells, cultured in conditions to mimic fatty liver disease^27^. The FASN inhibitor TVB-2640 reduced accumulation of cellular triglycerides induced by glucose/insulin stimulation (Fig. 1a). This was statistically significant at doses of 30 nM and 3 µM, of which the former approximates the cellular IC_50_ for inhibition of palmitate synthesis.

### FASNi treatment reduces expression of fibrotic and inflammatory markers

To directly assess the effect of FASN inhibition on HSC, TVB-3664 was incubated with the LX-2 human stellate cell line. Concentrations up to 150 nM of TVB-3664 were not cytotoxic (not shown). TVB-3664 inhibited proliferation of these cells, based on BrdU incorporation, in a dose-dependent manner with a modest effect at 150 nM (Fig. 1b). TVB-3664 (150 nM) decreased gene expression of *Col1α1* (− 68%), *αSMA* (− 60%), *βPDGFR* (− 54%), *TGFβR1* (− 53%), *TIMP1* (− 9%), *TIMP2* (− 24%), and *MMP2* (− 50%) in LX-2 cells (Fig. 1c). Gene expression was restored 48 h after removal of TVB-3664, indicating that the effect was not due to toxicity. Consistent with this, immunocytochemistry showed decreased Collagen 1α1 and αSMA proteins in LX2 cells (Fig. 1d). Similar decreases in protein expression of Collagen 1α1 and αSMA were observed in primary hHSCs (Fig. 1e), providing additional validation that FASN inhibition had a direct anti-fibrogenic impact.

The DNL pathway is important for Th17 cell production and function^21,22^, and therefore the effect of FASN inhibition was tested on the development of Th17 cells. CD4+ T cells isolated from blood of healthy volunteers were treated with a cytokine cocktail (IL-1β, IL-6, TGF-β and IL-23), that mimics the pro-inflammatory environment in the NASH liver to induce differentiation to Th17. As shown in Fig. 1f, these conditions generated ~ 30% Th17 cells in cells from two independent donors. FASN inhibition by TVB-3664 (50 nM) or TVB-2640 (100 nM) decreased development of Th17 cell population from human CD4+ T cells by > 75% or more, with Th17 cells ranging from 1.6 to 10.3%, while T regulatory cells were increased from < 1% (vehicle) to 20–30% in the presence of FASN inhibitor (Fig. 1f).

DNL also is necessary for inflammasome activation so the effect of FASN inhibition on secretion of inflammasome-mediated cytokines in human PBMCs was tested. To induce IL-1β secretion, cells were stimulated with either LPS to activate via toll-like receptor 4 (TLR4) or with lipotechoic acid to activate TLR2. Treatment with TVB-3166 for 48 h significantly reduced IL-1β secretion by more than 50% (Fig. 1g). Similar findings were observed across several independent human donors. Taken together, these in-vitro and ex-vivo results show that FASN inhibition acts directly on several cell types important for NASH.

### FASNi treatment prevents pro-inflammatory cytokine release and development of NASH in a diet-induced mouse model of NASH

**Figure 2:**
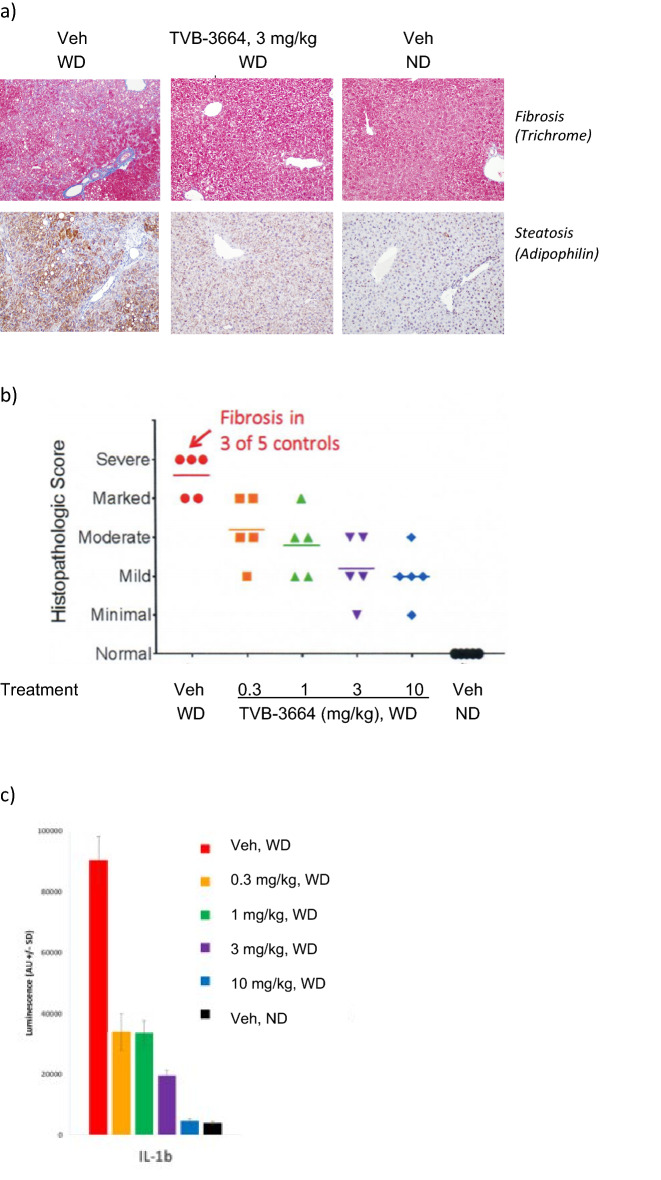
FASN inhibition prevents development of liver steatosis and fibrosis in a preventative NASH diet induced model. (**a**) Effect of FASNi on fibrosis and lipid accumulation. Liver sections at the end of study stained with Mason’s Trichrome or adipophilin, from vehicle or TVB-3664 (3 mg/kg) treated animals on a form of WD, or vehicle treated animals on a normal diet. Representative examples are shown. The scoring per animal is provided in Supplementary Table S1. (**b**) Histological scoring results of H&Estained liver sections. The mean of 5 animals is indicated by horizontal line. Scoring system: 0 (normal), 1 (minimal), 2 (mild), 3 (moderate), 4 (marked). The scoring per animal is provided in Supplementary Table S2. (**c**) IL-1β levels measured from serum collected at the end of the treatment period from vehicle or TVB-3664 treated animals.

TVB-3664 was tested in a series of mouse models of NASH. First, in a prevention model of diet induced NASH, C57BL/6J mice were fed a form of Western diet (WD), containing 40 kcal% fat (vegetable shortening), 20 kcal% fructose, and 2% cholesterol, and given vehicle or TVB-3664 at different dose levels orally once daily for 57 days, with 5 animals per group. TVB-3664 was well tolerated and food intake was similar in TVB-3664 and vehicle treated groups. Upon necropsy at the end of study, liver steatosis, as measured by adipophilin staining, and fibrosis, as measured by trichrome staining, were increased in vehicle treated animals on HFSD, and extensive tissue remodeling was evident (Fig. 2a). Cardinal morphologic features of livers of mice in this group were consistent with steatosis with early evidence of fibrosis and inflammation (Fig. 2a). In contrast, TVB-3664 (3 mg/kg) treatment was associated with decreased steatosis and fibrosis compared to the vehicle WD group (Fig. 2a and Supplementary Table S1). Histological scoring of H&E stained liver slides using a scale from normal (0) to severe (4) based on overall hepatic changes showed that TVB-3664 treatment was associated with a dose-dependent reduction in liver histology severity score (Fig. 2b and Supplementary Table S2), with a mean ± SD score of 4.6 ± 0.49 for the vehicle HFSD group, 3.2 ± 0.75 at 0.5 mg/kg and 2.0 ± 0.63 at 10 mg/kg TVB-3664.

Serum cytokine levels including IL-1β were elevated in the vehicle WD group in comparison to the vehicle standard diet control (Fig. 2c). TVB-3664 treatment resulted in a clear dose-related reduction in the level of IL-1β (Fig. 2c) and other cytokines (Supplementary Fig. S1). The highest dose levels tested of 3 or 10 mg/kg restored levels to those observed in vehicle control animals on a normal diet. The results from this preventative model indicate that FASN activity is required for the development of steatosis, pro-inflammatory activity, and fibrosis.

### FASNi treatment improves liver disease in a therapeutic diet-induced mouse model of NASH

**Figure 3:**
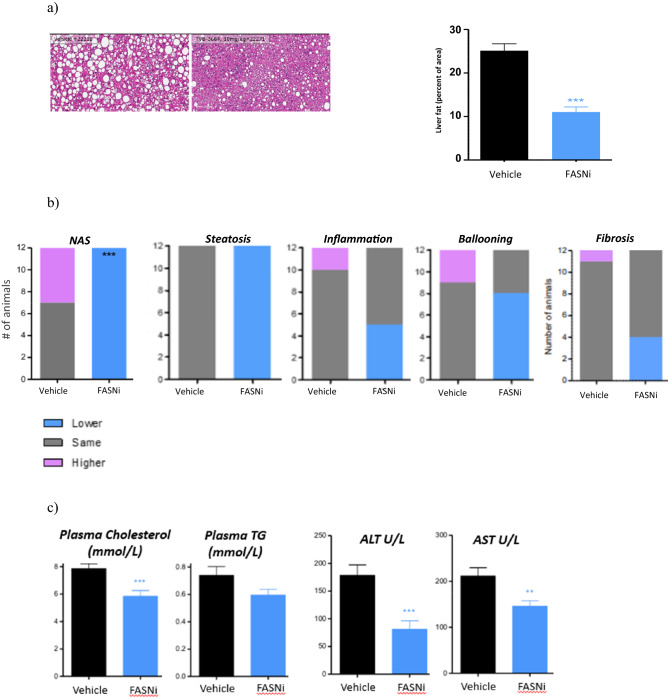
FASN inhibition reduces liver fat, metabolic parameters and NASH histology severity scores in a NASH diet induced therapeutic mouse model. (**a**) Effect of FASNi on liver histology. Left: Representative images of H&E stained liver sections at the end of the treatment period with vehicle or TVB-3664 (10 mg/kg). Magnification 10x, scale bar = 200 µm. Right: Liver fat (steatosis) determined by histological scoring. Mean ± SEM (n = 11/12 per group). ***p < 0.001 vs vehicle, One-Way ANOVA with Dunnett’s multiple comparison test. (**b**) Effect of FASNi on liver NAS score. Histological scoring of liver at the end of the treatment period. The number of animals with a higher (worse, pink), no change (grey) or lower (improved, blue) histology score in liver samples at end of study compared to prestudy biopsies for each animal is indicated by the height of the bar. ***p < 0.001 vs vehicle, Fisher’s exact test followed by adjustment for multiple correction using the Bonferroni method. (**c**) Effect of FASNi on plasma ALT, AST, cholesterol and triglyerides at the end of the treatment period. Mean ± SEM. **p < 0.01, ***p < 0.001 vs vehicle, One-Way ANOVA with Dunnet’s multiple comparison test.

TVB-3664 was next evaluated in a therapeutic (established disease) model, where diet-induced NASH was established prior to initiation of drug treatment. C57BL/6J male mice were fed a form of WD containing 40 kcal% fat (vegetable shortening), 20 kcal% fructose, and 2% cholesterol for 44 weeks to induce obesity, steatohepatitis and fibrosis. A pre-treatment liver biopsy was performed to confirm the presence of NASH with fibrosis, and animals were then treated once-daily with either TVB-3664 at 10 mg/kg or placebo for the next 8 weeks (n = 11/12 per group). TVB-3664 was well tolerated and food intake was similar in TVB-3664 and vehicle treated groups. Upon necropsy, histological scoring of livers was performed using the NAFLD Activity Score (NAS) CRN method^28^. TVB-3664 treatment reduced total lipid content and % area of the liver (p < 0.001 vs vehicle) (Fig. 3a). Of the 3 NAS components, steatosis improved in 100% of animals, ballooning in ~ 40% and inflammation in 66% of animals, respectively. Overall, TVB-3664 reduced NAS score in 100% of animals from a mean score of 6 pre-treatment to 3.8 post-treatment (p < 0.001). There was no improvement in any vehicle-treated mice, with a mean NAS score of 6.5 both pre and post-treatment (Fig. 3b). Moreover, TVB-3664 treatment was associated with lowering liver fibrosis stage in approximately 30% of animals (Fig. 3b).

Treatment with TVB-3664 at 10 mg/kg significantly reduced plasma ALT and AST levels compared to vehicle treatment (Fig. 3c). Plasma triglycerides and plasma cholesterol levels were also reduced (Fig. 3c). TVB-3664 also reduced liver weight, liver triglycerides and liver cholesterol levels (Supplementary Fig. S3). These results show that 8 weeks of FASN inhibitor treatment reduced liver NAS score and fibrosis stage in this established disease setting, and was associated with improved markers of liver and metabolic health.

### FASNi treatment attenuates NASH and hepatocellular carcinoma in the FAT-NASH (CCl_4_) mouse model with advanced fibrosis

The effect of FASN inhibition was also evaluated in a therapeutic model of diet-induced NASH with CCl_4_ administration to cause liver fibrosis, recently termed the FAT (Fibrosis And Tumors)-NASH model^29^. C57BL/6J mice were fed a form of WD, containing 21.2% fat (42% Kcal), 41% sucrose and 1.25% cholesterol WD and sugared water, and administered once weekly CCl_4_ for 12 weeks to induce NASH with liver damage. Then oral TVB-3664 (5 mg/kg, n = 8) or vehicle (n = 9) was added once daily to the weekly CCl_4_ for an additional 12 weeks. TVB-3664 was well tolerated and food intake was similar in TVB-3664 and vehicle treated groups.

**Figure 4:**
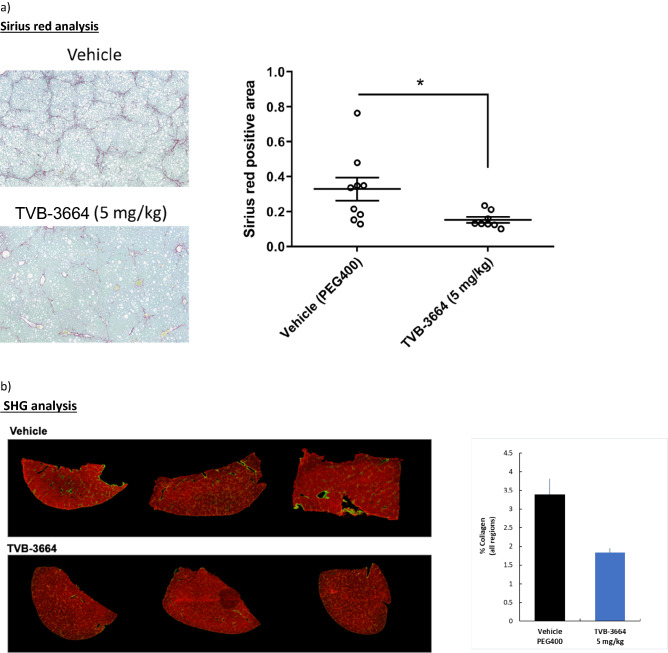

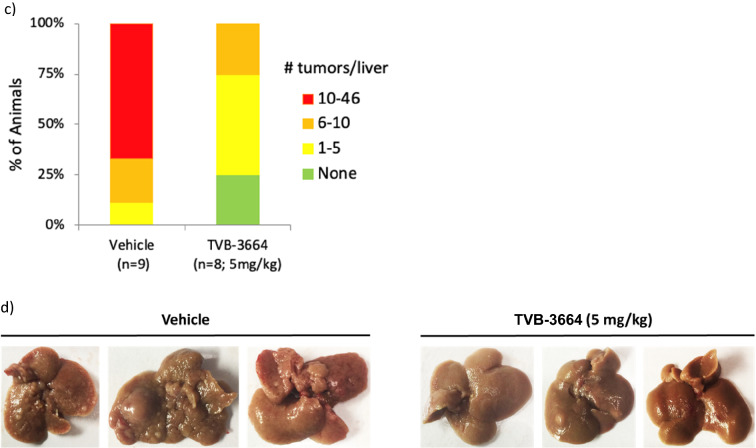
FASN inhibition reduces established fibrosis and hepatocellular carcinoma tumor formation in the FAT-NASH (CCl_4_) Model. (**a**) Effect of FASNi on liver fibrosis by conventional pathology. Left: Photomicrograph of representative sirius red/fast green stained liver sections from vehicle or TVB-3664 treatment (5 mg/kg). Right: Quantification of fibrillar collagen using BioQuant software. *p < 0.0 vs vehicle, unpaired two tailed Mann–Whitney test. (**b**) Effect of FASNi on liver fibrosis by AI based digital pathology. Quantification of total collagen area using Second Harmonic Generation. Left: Representative images from vehicle and TVB-3664 treated. Collagen is represented in green. Right: Graph shows mean/SD of all animals. An expanded view of representative images is shown in Supplementary Fig. S2. (**c**) Effect of FASNi on the development of hepatocellular carcinoma. Number of liver tumors per animal at the end of treatment with vehicle or TVB-3664. (**d**) Effect of FASNi on appearance of livers at the end of treatment with vehicle or TVB-3664. Representative images are shown.

At 24 weeks, vehicle treated animals showed advanced bridging fibrosis, ballooning and inflammation. In contrast TVB-3664-treated mice had significantly reduced fibrosis (Fig. 4a), with regression of fibrillar collagen content by 53% versus vehicle control (Fig. 4a). As assessed by AI-based second harmonic generation (SHG) analysis, animals receiving TVB-3664 had decreased liver collagen content (Fig. 4b). Hepatocellular carcinoma (HCC) developed in all vehicle treated mice, with 4–46 liver tumors per animal. The number of liver tumors was reduced overall by 85% by TVB-3664 (p < 0.05) (Fig. 4c). Representative liver pictures after necropsy are shown in Fig. 4d. At the end of study, liver weight ratios were not significantly different across the groups (data not shown).

**Figure 5:**
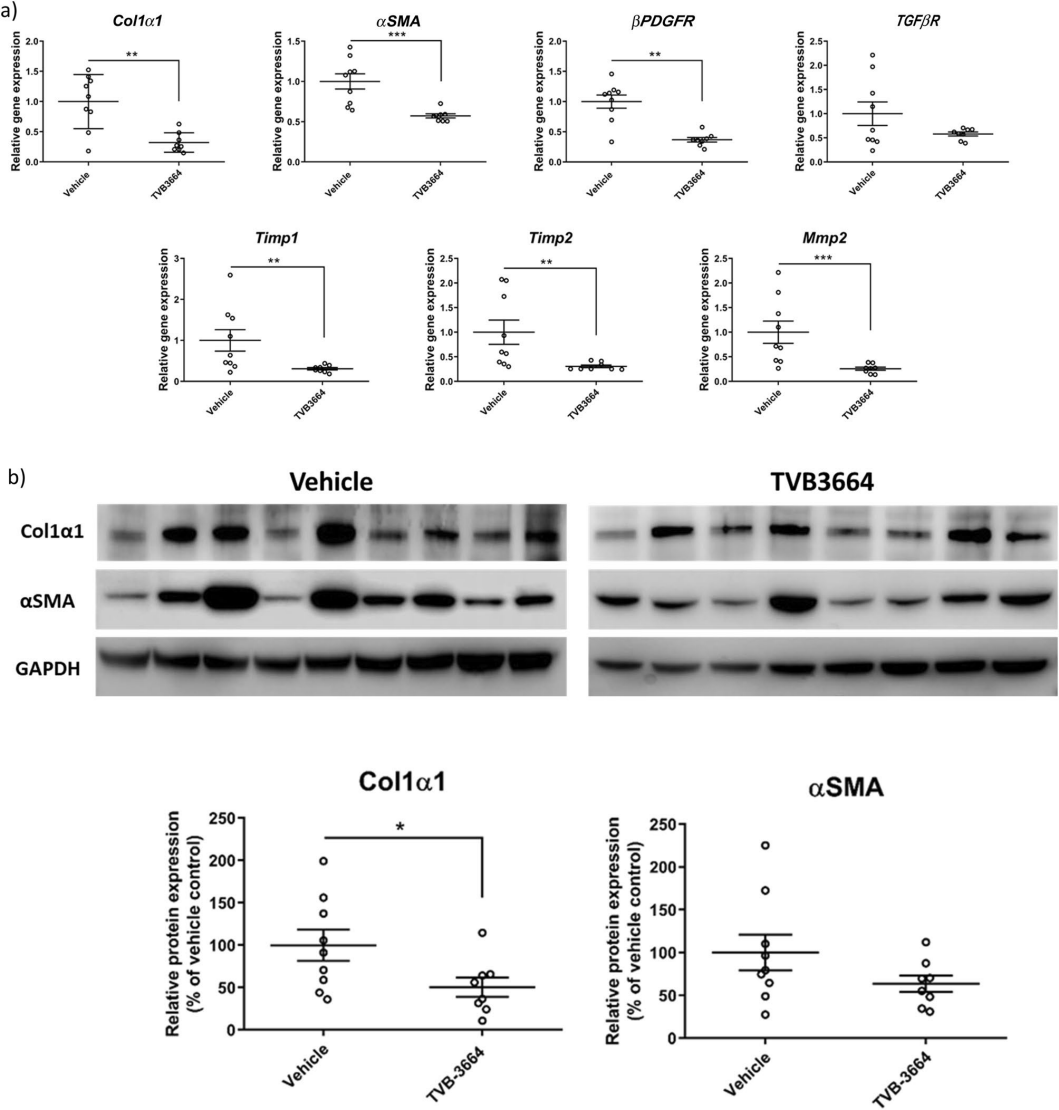

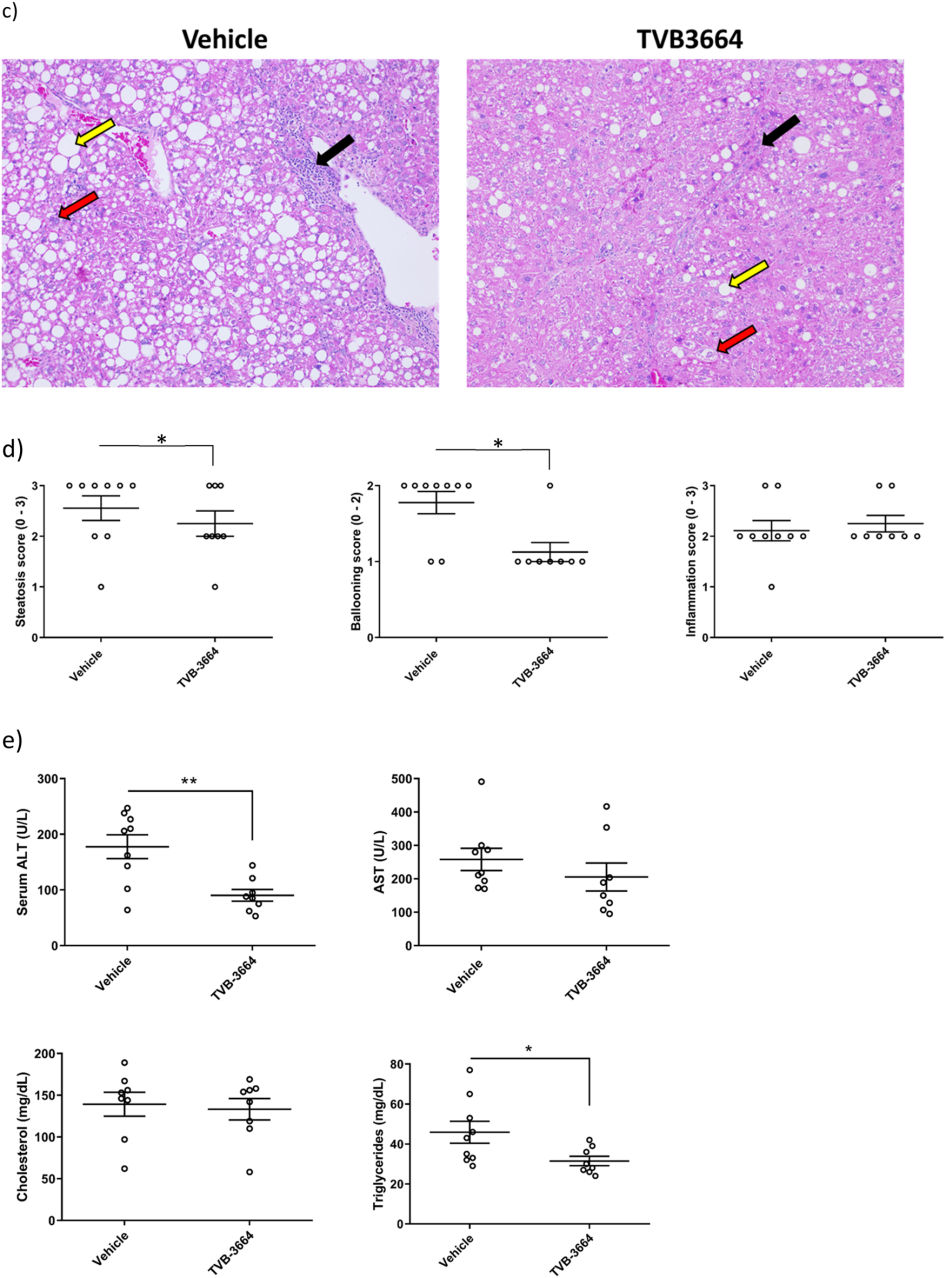
Gene and protein expression, and NAS score in the FAT-NASH (CCl_4_) Model. (**a**) Effect of FASNi on mRNA expression of fibrogenic genes. liver tissues measured by RT-qPCR treated with vehicle or TVB-3664 (5 mg/kg). (**b**) Effect of FASNi on protein expression of fibrogenic genes. Western blot analysis of protein expression of Col1A1 and αSMA in liver tissue from animals treated with vehicle or TVB-3664. (**c**) Effect of FASNi on liver histology by H&E. Representative photomicrographs of H&E stained liver sections in vehicle (PEG400) and TVB-3664. Steatosis (yellow arrow), hepatocyte ballooning (red arrow) and lobular inflammation (black arrow) are indicated. Statistical analysis was by unpaired two tailed Mann–Whitney test. *p ≤ 0.05 vs vehicle group. (**d**) Effect of FASNi on liver histology by NAS score Analysis performed blinded at the end of treatment with vehicle or TVB-3664. Lines represent Mean/SD. (**e**) Effect of FASNi on plasma AST, ALT, total cholesterol and triglycerides. Lines represent Mean/SD. Statistical analysis was by unpaired two tailed Mann–Whitney test. *p ≤ 0.05 vs vehicle group, **p ≤ 0.01 vs vehicle group, ***p ≤ 0.001 vs vehicle group.

To gain insight into the mechanism of action, we assessed a panel of fibrogenic genes in liver samples. There was a significant reduction of Col1α1 (p = 0.005), α smooth muscle actin (αSMA) (p = 0.0003), PDGFRβ (p = 0.002), TIMP-1(p = 0.007), TIMP-2 (p = 0.003), and MMP-2 (p = 0.001) in mice treated with TVB-3664 (Fig. 5a). Col1α1 and αSMA protein expression were decreased in TVB-3664-treated compared to vehicle treated animals by 49% (p = 0.04) and 31% (p = 0.23), respectively (Fig. 5b, and Supplementary Fig. S4). TVB-3664 treatment was associated with significantly decreased steatosis and hepatocyte ballooning compared to vehicle controls, as seen in H&E staining (Fig. 5c,d). Lobular inflammation was similar in the groups and most animals had a score of 2 (Fig. 5d). TVB-3664 treatment led to significant decreases in serum ALT (49%, p = 0.007) and AST decreased by20% (Fig. 5e). A 31% decrease in serum triglycerides was observed in TVB-3664 compared to vehicle treated animals (p = 0.02). These results show that FASN inhibition improved established liver disease and prevented the development of HCC in a NASH model with aggressive fibrosis.

## Discussion

NASH is a multi-faceted disease involving several cell types and mechanisms. Lipid accumulation in hepatocytes leads to steatosis and generation of lipotoxins, which potentiates inflammation and drives fibrogenesis in stellate cells. FASN plays a role in several distinct cell types in NASH and impacts each of these three core mechanisms. FASN inhibition therefore has potential to target multiple aspects of NASH pathogenesis. FASN’s role in lipid synthesis is well established, and the FASN inhibitor TVB-2640 decreases hepatic DNL in healthy volunteers with characteristics of metabolic syndrome^30^ and liver fat in NASH patients^24^.

This is the first manuscript describing evaluation of this class of potent and selective FASN inhibitors in preclinical NASH models. This provides important proof of concept for FASN inhibition and contributed to nomination of TVB-2640 (denifanstat), a first in class FASN inhibitor for clinical development in NASH.

In this series of studies, in vitro and ex-vivo models were used to directly test the effect of FASN inhibition on inflammation and fibrosis in human culture models of primary LMT, the LX-2 HSC cell line, primary hHSC, CD4+ T cells and PBMC. The ex-vivo human LMT model demonstrated that FASN inhibition decreases cellular triglycerides, a marker of steatosis or liver fat accumulation. Furthermore, FASN inhibition also directly reduced fibrogenic activity of primary hHSCs and pro-inflammatory activity in human immune cells. In vivo, this translated to efficacy in mouse models of diet induced NASH, evidenced by decreased steatosis, inflammation, and fibrosis and reduced development of hepatocellular carcinoma. In vivo efficacy was tested in three murine models of NASH; a prevention model of diet-induced NASH, a therapeutic model of diet-induced NASH, and a therapeutic FAT-NASH model with aggressive liver fibrosis injury that develops hepatocellular carcinoma.

FASN inhibition in both the LX-2 stellate cell line and primary hHSC reduced the expression of genes involved in stellate cell activation, including Col1α1, αSMA, βPDGFR, and decreased collagen and αSMA protein expression, indicating that FASN inhibition directly reduces HSC fibrogenic activity. Acetyl-CoA carboxylase (ACC) inhibitors, one step upstream from FASN in the DNL pathway, have shown similar effects; the ACC inhibitor PF05221304 inhibits DNL in primary HSC^9^ and the ACC inhibitor firsocostat/GS9076 and a FASN inhibitor decreased TGF-beta induced DNL and procollagen1 production in LX-2 cells^8^. Collectively, these findings from multiple independent studies, including the current study, using different chemical scaffolds demonstrate that DNL inhibitors directly decrease HSC activation, consistent with decreased formation of activated myofibroblasts, which is important for HSC-mediated fibrosis^10^. This effect may be mediated by preventing the activation of a NLRP3 structure similar to the inflammasome, although the specific steps remain to be elucidated^18,19,31^.

FASN inhibition decreased several measures of inflammation in primary human immune cells. A likely mechanism for the decreased IL-1β production in PBMC is that decreased palmitate production resulting from FASN inhibition leads to decreased NLRP3 inflammasome activation^18,19^. The decrease in Th17 cell production in the presence of FASN inhibitors is consistent with results from Young et al., with a different FASN inhibitor C75, which showed that FASN is required for production and differentiation of Th17 cells^22^. Similar results have been found for ACC inhibitors^9,21^, and a proposed mechanism is that the DNL pathway is required for metabolism and/or membrane production in the highly proliferating and signaling Th17 cells but not in Treg cells. The development of hepatic inflammation and lipotoxicity potentiates the development of fibrosis in NASH. Taken together, these primary human cell data indicate that FASN inhibition directly attenuates pathways of fibrogenesis and inflammation relevant to NASH, as well as reducing steatosis.

Since no single animal model of NASH is fully predictive of drug efficacy in humans, we explored FASN inhibition in three separate mouse diet-induced models spanning the spectrum of disease severity; a prevention model, a therapeutic model, and a therapeutic model with advanced fibrosis and tumor formation (FAT-NASH). Results were consistent across these models. FASN inhibition decreased ALT, AST levels consistent with decreased liver injury. Circulating TG levels were tested in the therapeutic and FAT-NASH models and decreased slightly with FASNi, different to the increased TGs reported for ACC inhibitors in some NASH models^9,32^ and ACC inhibitors in clinical studies^33,34^. This could be due to potentially different impact of ACCi and FASNi on malonyl-CoA levels and subsequent lipid pathways, and indicates that targeting different nodes on the DNL pathway may have different downstream effects. Across all 3 models with FASNi, liver histology showed decreased steatosis and improved liver histology, which was associated with decreased NAS score measured using CRN criteria in the therapeutic and FAT-NASH model. In the severe FAT-NASH model, fibrosis and collagen content were significantly decreased by TVB-3664, consistent with the in vitro findings of decreased fibrogenic activity in HSC. This is the first report of a DNL inhibitor in this model. In this model, fibrosis was measured by both conventional histology and using an artificial intelligence (AI)-based digital SHG platform, with consistent results. Use of AI-based approaches are increasingly incorporated into clinical NASH studies to complement traditional histology, because AI-based approach can more accurately quantify fibrosis on a continuous scale and establish co-localization with other parameters including steatosis. Both approaches will be incorporated into the Ph2b NASH study with TVB-2640. Overall, these results indicate that FASN inhibition alleviates several hallmarks of NASH in this aggressive fibrosis model.

The FASN inhibitor effects in the severe FAT-NASH model reduced development of HCC tumors, a clinical risk associated with severe NASH. It has recently been shown that TVB-3664 suppresses AKT mediated hepatic steatosis in a mouse HCC model and that specific oncogene-driven subsets of HCC such as the MET-high/PTEN low are particularly FASN-dependent^35^. The ability of FASN inhibition to reduce the emergence of hepatocellular carcinoma in mice with significant liver injury provides the potential for further evaluation of this treatment for this devastating disease. Mechanisms underlying HCC inhibition by FASN inhibitors merit further studies to distinguish between indirect effects by reducing the metabolic, inflammatory and fibrogenic drivers of tumorigenesis and direct effects on early tumor cells. Our preclinical results show that FASN inhibitors act directly on the 3 major cell types driving NASH pathogenesis: hepatocytes, inflammatory cells and HSCs. Specifically, FASN inhibition reduces intracellular triglyceride levels in LMT, reduces cytokine expression from inflammatory cells, blunts the induction of pro-inflammatory Th17 cells and reduces the fibrogenic potential of phHSCs. In vivo, FASN inhibition had a multi-faceted impact on liver damage across different models reducing liver fat, inflammatory markers, and fibrotic scar tissue. In addition, many of the other sequelae often present in NASH patients and animal models of NASH including elevated plasma ALT and triglycerides were improved by FASN inhibitor treatment. This may result from a direct effect of FASN inhibition on these major cell types as well as an indirect effect whereby FASN inhibition of the instigating event of fat accumulation in hepatocytes leads to the removal of the downstream lipotoxic damage elicited by excess hepatic fat.

Denifanstat is in clinical development for NASH. In a 12-week Phase 2a study FASCINATE-1, denifanstat reduced liver fat content in subjects with NASH and improved serum markers of fibrosis and inflammation, including ALT, PRO-C3 and CK-18^24^. These results are consistent with the nonclinical efficacy and mechanistic data described in this study, indicating that FASN inhibitors act directly on the major cell types of NASH pathogenesis.

## Materials and methods

### Compounds

TVB-2640 (denifanstat), TVB-3166 and TVB-3664 were designed and synthesized by Sagimet Biosciences (formerly 3-V Biosciences), and have been previously described^24–26,36^. For in vivo studies, TVB-3664 was administered once daily by oral gavage formulated in 30% PEG400 in water. For in vitro studies, compounds were formulated as stock solutions in DMSO and diluted prior to use.

### Cell based assays

#### Biochemical and cellular potency assays

Conducted at 3-V Biosciences, Menlo Park, CA.

FASN enzymatic activity was determined using a fluorescence-based thiol quantification assay as described by Chung et al.^37^. The assay monitors the release of Coenzyme A (CoA), a byproduct of FASN activity, which reacts with the profluorescent dye CPM (7-Diethylamino-3-(4-maleimidophenyl)-4-methylcoumarin) (Sigma C1484) and makes it fluorescent. For FASN biochemical assays, human FASN protein was extracted from SKBr3 cells. Palmitate synthesis assays were performed as previously described^25^, by cell incubation in media containing ^13^C-acetate and measurement of conversion to palmitate by LC–MS analysis. The mean of 2 to 15 independent assays are shown. Data were analyzed using Excel (Microsoft Corp., Redmond, WA) and Prism (GraphPad Software, Inc., La Jolla, CA) software.

#### LMT

Conducted by the Contract Research Organization Insphero AG (Switzerland). LMTs were created using cryopreserved primary human cells and cultured in 96 well Akura plates using established protocols, as previously described^27^ Total TG content was measured with the TG-Glo assay (Promega, J3161).

#### LX-2

Conducted by the Friedman lab. The procedures were followed as previously described^29^. Briefly, in 6-well culture plate 150,000 LX-2 cells were seeded per well and serum starved overnight. Two sets of culture plates were run in parallel. Both sets of LX-2 cells were induced with either vehicle (DMSO) or indicated dosing of TVB-3664. One set of LX-2 cells were harvested after 48 h of TVB-3664 treatment before harvest. In other set of cells both vehicle and TVB-3664 were replaced by normal DMEM culture media with 10% FBS and maintained for additional 48 h before harvest. For RNA expression, total RNA was extracted from harvested LX-2 cells, cDNA was synthesized and expression of fibrogenic genes was quantified by qPCR. Unpaired T tests were used to compare the mean results per group appropriate since these were independent groups.

#### Immunocytochemistry of HSCs

Conducted by the Friedman lab. Primary human hepatic stellate cells (phHSCs) were isolated, purified and cultured from discarded remnants of surgically resected human livers of de-identified patients. The protocol was reviewed and approved by Institutional Review Board (IRB) of Icahn School of Medicine at Mount Sinai, NY, and informed consent was obtained. All methods were performed in accordance with the Declaration of Helsinki. Col1α1 and αSMA either in LX-2 or phHSCs in presence of TVB-3664 small molecule were determined by immune-staining DAB technique. 100,000 LX-2 cells or 80,000 primary hHSCs were seeded on glass coverslip. Cells were starved overnight in DMEM supplemented with 0.1% BSA (without antibiotic) to synchronize metabolic activities of the cells. Cells were then incubated with either vehicle or TVB-3664 FASNi at the indicated concentration and duration. The cells were washed thoroughly with 1× PBS and fixed in 4% paraformaldehyde, permeabilized with 0.5% Tween-20 in 1× PBS and blocked in Dako peroxidase block (0.03% H_2_O_2_, sodium azide; Agilent Technologies, CA). To avoid non-specific antibody binding the cells were re-block with Dako protein block serum-free reagent (Agilent Technologies, CA). The cells were immunostained with rabbit anti-Collagen1 (Rockland Immunochemicals Inc., PA) or rabbit anti-αSMA (Abcam, MA) primary antibodies for overnight. A set of no primary antibody control cell was run in parallel as background control (data not shown). The secondary antibody used for this study was Dako labelled polymer-HRP anti-rabbit (Agilent Technologies, CA) and incubated for 1 h. The cells were then treated with Dako DAB-chromogen (Agilent Technologies, CA). Nuclear counter staining was performed with hematoxylin (Sigma-Aldrich, MO). Antibody signals was captured with Axiocam 503 mono camera (Zeiss, NY) using 10× objective in an AxioImager Z2 upright microscope (Zeiss, NY). Image acquisitions were analyzed by Zen2 software (Zeiss, NY).

#### PBMC and CD4+ T cells

Conducted by Sagimet Biosciences (formerly 3-V Biosciences). Healthy human donor blood collected in sodium citrate tubes was purchased from The Stanford Blood Center, Palo Alto, CA. Appropriate informed consent and IRB approval was obtained under licenses in place at Stanford Blood Center (https://stanfordbloodcenter.org/research-updates/donating-for-research/). All samples were de-identified. All methods were performed in accordance with the Declaration of Helsinki. Freshly collected PBMC were isolated from by Ficoll Paque Plus density gradient separation (Millipore Sigma, GE17-1440-02). Naïve CD4+ T cells were enriched with Dynabeads™ human CD4+ T Cells Isolation Kits (Thermo Fisher, Cat# 11346D).

Naïve human CD4+ T cells were then differentiated by plating into 48-well tissue culture plates (Costar) with 1 μg/mL anti-CD3 with anti-CD28 antibody coated beads. Differentiation to generate human Th17 naïve human CD4+ T cell cultures were placed in medium containing the following additives for 4 days: anti-IL-2 (30 ng/mL, anti-IL-4 antibody (20 ng/mL), anti-IFNγ antibody (50 ng/mL) from R&D Systems, recombinant human IL-6 (10 ng/mL), human TGFβ (1 ng/mL) (recombinant human IL-1β (10 ng/mL) recombinant human IL-23 (10 ng/mL) from BD Biosciences. The resulting Th17 cells were cultured in IMDM GlutaMAX medium (Life Technologies) supplemented with 10% heat-inactivated FCS (Life Technologies) with 500 units of penicillin–streptomycin (Life Technologies). IL-1β from primary human PBMC supernatant was measured by ELISA according to manufacture’s instructions (DLB50, R&D Systems).

#### Flow cytometry

Conducted by Sagimet Biosciences (formerly 3-V Biosciences). Monoclonal antibodies specific to the following antigens (and labeled with the indicated fluorescent markers) were used: Foxp3 FITC (236A/E7; 1:100), (Affymetrix/eBioscience), IL-17A PE-Cy7 (eBio64DEC17; 1:100). (Affymetrix/eBioscience), CD4 PerCP (SK3; 1:200) (BD Biosciences).

For analysis of surface markers, cells were stained in PBS containing 0.25% BSA and 0.02% azide. Dead cells were excluded by LIVE/DEAD Fixable Dead Cell Stain Kit (Life Technologies). For intracellular cytokine staining cells were treated with Brefeldin A (5 mg/mL) for 2 h and stained using the Fixation/Permeabilization Kit (BD Biosciences) according to the manufacturer’s instructions. Quantitation of fluorescent cells and their staining intensity were acquired on a FACSCalibur (BD Biosciences), and data were analyzed with CELLQuest software.

### Mouse models

All animal housing and research procedures and protocols involving live animals were approved by respective local Institutional Animal Care and Use Committee (IACUC). Animal welfare for the studies described was in compliance with the U.S. Department of Agriculture’s (USDA) Animal Welfare Act (9 CFR Parts 1, 2, and 3). All studies were conducted in accordance with ARRIVE guidelines. Additional detail on statements of approval, licenses, animal care, housing and euthanasia for each vertebrate study are provided in the Supplemental Methods file. In all three models animals were fed a diet designed to mimic a Western diet (WD) containing high fat, high sugar plus cholesterol.

#### Preventative NASH diet-induced model

In life conducted by the Contract Research Organization Care Research (Fort Collins, CO) using 5-week old male C57BL/6J mice. CARE is a USDA certified (Certificate Number 84-R-0081) and OLAW accredited facility. The study design and animal usage were reviewed and approved by the CARE Research IACUC for compliance with regulations prior to study initiation (IACUC number 1628). Mice were fed a diet designed to imitate a WD, containing 40 kcal% fat (vegetable shortening), 20 kcal% fructose, and 2% cholesterol (#D09100301, Research Diets, Inc.), or normal standard commercial rodent diet (Teklad Rodent Chow 2018,Harlan). Liver samples were processed and histology performed at IDEXX BioResearch (Sacramento). Masson’s Trichrome stain was used to assess the amount of fibrous connective tissue in the liver, and Oil Red O and adipophilin stained sections were used to characterize histologic features associated with steatosis. A qualified pathologist (Tox Consult LLC) performed histologic evaluation using the following scoring system on H&E stained sections: 0 = normal; 1 = minimal changes; 2 = mild changes; 3 = moderate changes; 4 = marked changes and 5 = severe changes. Cytokine/chemokine assays were performed by Sagimet Biosciences using terminal bleed samples, by ELISA according to manufacturers instructions (EA-4003, Signosis Inc).

#### Therapeutic NASH diet-induced model

Conducted by the Contract Research Organization Gubra (Denmark) using 5-week old male C57BL/6J mice. All animal experiments were conducted in accordance with Gubra’s bioethical guidelines, fully compliant to internationally accepted principles for the care and use of laboratory animals. The experiments were covered by licenses for Jacob Jelsing (2013-15-2934-00784 and 2015-15-0201-00518) issued by the Danish committee for animal research. Mice were fed a diet designed to imitate a WD, containing 40% high trans-fat with high sugar (20% fructose, 9% sucrose) and 2% cholesterol (Research Diets, Inc., #D09100310), for 44 weeks prior to treatment with TVB-3664, and during treatment. Blood samples were collected in heparinized tubes and plasma separated and stored at -80 °C until analysis. TG, TC, ALT, AST were measured using commercial kits (Roche Diagnostics, Germany) on the Cobas TM C-501 autoanalyzer according to the manufacturer’s instructions. Liver IHC used standardized procedures. NAS scoring used the criteria outlined in Kleiner et al.^28^. Fibrosis was scored on the basis of 0 (none), 1 (perisinusoidal or periportal), 2 (perisinusoidal and portal/periportal), or 3 (bridging fibrosis). Statistical analysis used one-way ANOVA with Dunnett’s multiple comparison test to compare treated to vehicle. For change in histology score per animal, Fisher’s exact test followed by adjustment for multiple correction using the Bonferroni method.

#### Fibrosis and tumor (FAT)-NASH CCl4 model

Conducted by the Friedman group using 6-week old male C57BL/6J mice. This animal protocol was approved by the IACUC at the Icahn School of Medicine at Mount Sinai, NY (IACUC-2015-0112). The model has been previously described^29^. Mice were fed a diet designed to imitate a WD, containing 21.2% fat (42% Kcal), 41% sucrose and 1.25% cholesterol (Teklad Custom diet Cat# TD.120528, Envigo, WI), and access to sugar water solution (18.9 g/L d-(+)-Glucose (Sigma-Aldrich, MO) and 23.1 g/L d-(−)-Fructose (Sigma-Aldrich, MO), and once weekly CCl4 IP injection (0.2 µ/g) for 24 weeks. Mice were treated with either 3 mg/kg TVB-3664 or vehicle (PEG400) for the last 12 weeks, after developing NASH and hepatic fibrosis. Blood serum analysis, fibrogenic gene and protein expression, collagen quantification by Bioquant and NAFLD activity score (NAS) were analyzed as previously described^29^. Data analysis was accomplished by using GraphPad Prism v7.4 statistical software (GraphPad Software, Inc., CA). Standard error mean (± SEM) was calculated according to unpaired two tailed Mann–Whitney test where Gaussian distribution is non-parametric. Unless otherwise specified, p values < 0.05 were considered statistically significant.

AI-based SHG analysis was conducted by HistoIndex (Singapore) using established protocols as previously described^38^. Liver samples were from the same blocks as used for collagen quantification by routine histology. This AI-based approach uses two-photon excitation imaging coupled with second harmonic generation (SHG) imaging and identifies collagen and the surrounding structures directly from unstained tissue slides (formalin-fixed and paraffin-embedded)^33,34^.

## Supplementary Information

Supplementary Information 1.

Supplementary Information 2.

## Data Availability

The datasets generated and analyzed in these studies are available from the corresponding author upon request.
