# Supplementary material for: FASN inhibition targets multiple drivers of NASH by reducing steatosis, inflammation and fibrosis in preclinical models
Source: Sci Rep. 2022 Sep 19;12:15661. doi: 10.1038/s41598-022-19459-z (PMC9485253; doi:10.1038/s41598-022-19459-z)
Supplement: Supplementary file 1 — Supplementary Information 1. [file 41598_2022_19459_MOESM1_ESM.docx]

**O’Farrell et al.**

**Supplemental Methods**

**Animal Studies: methods of anesthesia and/or analgesia, housing and health monitoring.**

All studies were conducted in accordance with IACUC approval and local regulations. All studies were conducted in accordance with ARRIVE guidelines.

FAT-NASH (CCl_4_) mouse model (conducted at the Friedman Laboratory, Mt Sinai, NY).

- (1) Methods of sacrifice: Under deep anesthesia the abdomen of each animal was opened and blood samples were collected through the inferior vena cava for serum isolation to assay liver enzymes and lipid levels. Liver and spleen were collected and their weights were recorded. Tumor numbers from liver were recorded. mRNA and protein quantification and histology of the liver were performed on small excised pieces of the left lobe.
- (2) Methods of anesthesia and/or analgesia: The mice were anesthetized by Ketamine-Xylazine via IP injection and deep anesthesia confirmed by pedal reflex (firm toe pinch).
- (3) Efforts to alleviate suffering: To minimize suffering, surgical anesthesia was induced by intraperitoneal injection of ketamine/xylazine and monitored by loss of toe pinch response.
- (4) Basic housing and health monitoring: The animal protocol was approved by the Institutional Animal Care and Use Committee (IACUC) at the Icahn School of Medicine at Mount Sinai, NY (IACUC-2015-0112). Six week old C57BL/6J mice were housed (five mice/cage) in a Helicobacter-free room for 12 h light and 12 h dark cycles and weighed once per week. Animal health conditions were monitored every week by body condition score (BCS) by monitoring their activity levels during the entire period of study to ensure there was no toxicity or reduced activities as a result of drug administration. Drug or vehicle treatment was started on week 13 and continued up to week 24.

Diet induced mouse model of NASH (conducted by Contract Research Organization, Gubra, Denmark)

- (1) Methods of sacrifice: Animals were anesthesized using isofluorance gas, followed by cardiac puncture for collection of terminal plasma, whereafter animals were terminated by cervical dislocation.
- (2) Methods of anesthesia and/or analgesia: The mice were anesthetized with isoflurane for collection of liver biopsies or blood samples. For liver biopsy collection, during anesthesia with isoflurane, the abdominal cavity was opened and cardiac blood drawn with a regular syringe into EDTA tubes or with a coated (heparin/EDTA) vacutainer. Liver biopsy: Mice were anesthetized with isoflurane (2-3%) in atmospheric air. A small abdominal incision was made in the midline and the left lateral lobe of the liver is exposed. A cone shaped wedge of liver tissue (approximately 50 mg) was excised from the distal portion of the lobe and fixated in 10% neutral buffered formalin (4% formaldehyde) for histology. The cut surface of the liver was instantly electrocoagulated using bipolar coagulation (ERBE VIO 100 electrosurgical unit). The liver is returned to the abdominal cavity, the abdominal wall was sutured and the skin closed with staplers. For post-operative recovery mice received carprofen (5mg/kg) administered subcutaneously on operation day and on days 1 and 2 post-operation.
- (3) Efforts to alleviate suffering: Animals received at least daily monitoring and health checks, and post-operative care after liver biopsy as described above.
- (4) Basic housing and health monitoring: Animal experiments were conducted in accordance with Gubra’s bioethical guidelines, which are fully compliant to internationally accepted principles for the care and use of laboratory animals. The experiments are covered by personal licenses for Jacob Jelsing (2013-15-2934-00784 and 2015-15-0201-00518) issued by the Danish committee for animal research. The animals were checked a minimum once daily. Health status judged to warrant additional evaluation was to be examined by a Clinical Veterinarian, or a technician working under the supervision of the Clinical Veterinarian. The animal room environment was controlled (targeted ranges: temperature 21 ± 2 ̊C; relative humidity 50 ± 10%).

Diet induced mouse model of NASH (conducted by Contract Research Organization, Care Research, Fort Collins, CO)

- (1) Methods of sacrifice: Euthanasia was conducted via exsanguination under Isoflurane anesthesia in accordance with accepted American Veterinary Medical Association (AVMA) guidelines.
- (2) Methods of anesthesia and/or analgesia: The mice were anesthetized with isoflurane for collection of samples.
- (3) Efforts to alleviate suffering: To minimize suffering, animals received at least daily monitoring and health checks.
- (4) Basic housing and health monitoring: Detailed clinical observations were conducted at least once daily, with additional cage-side observations conducted at least once daily. Food consumption and body weights were recorded twice each week. During acclimation and live-phase, all mice were single-housed in polycarbonate shoe-box cages with an appropriate bedding material. Housing conformed to standards set forth in the Guide for the Care and Use of Laboratory Animals Environmental controls were set to maintain temperatures from 64o to 79oF. Temperature was monitored electronically and recorded daily. Humidity was maintained from 20% to 70%.
- All procedures used were conducted in accordance with all state, local, and federal laws and regulations, and abided by USDA guidelines for animal care and handling. All relevant CARE Research, LLC SOPs and IACUC requirements were followed in the execution of this study. CARE is a USDA certified (Certificate Number 84-R-0081) and OLAW accredited facility. All procedures used by CARE personnel during and for the conduct of this study were described in approved Standard Operating Procedures (SOPs) that were maintained at the study site and were available to all applicable laboratory personnel. The study design and animal usage were reviewed and approved by the CARE Research Institutional Animal Care and Use Committee (IACUC) for compliance with regulations prior to study initiation (IACUC number 1628). Animal welfare for this study was in compliance with the U.S. Department of Agriculture’s (USDA) Animal Welfare Act (9 CFR Parts 1, 2, and 3), the Guide for the Care and Use of Laboratory Animals, and CARE Research SOPs.
