# Supplementary material for: FASN inhibition targets multiple drivers of NASH by reducing steatosis, inflammation and fibrosis in preclinical models
Source: Sci Rep. 2022 Sep 19;12:15661. doi: 10.1038/s41598-022-19459-z (PMC9485253; doi:10.1038/s41598-022-19459-z)
Supplement: Supplementary file 2 — Supplementary Information 2. [file 41598_2022_19459_MOESM2_ESM.docx]

**Supplementary Figures and Table**

**Fig S1**


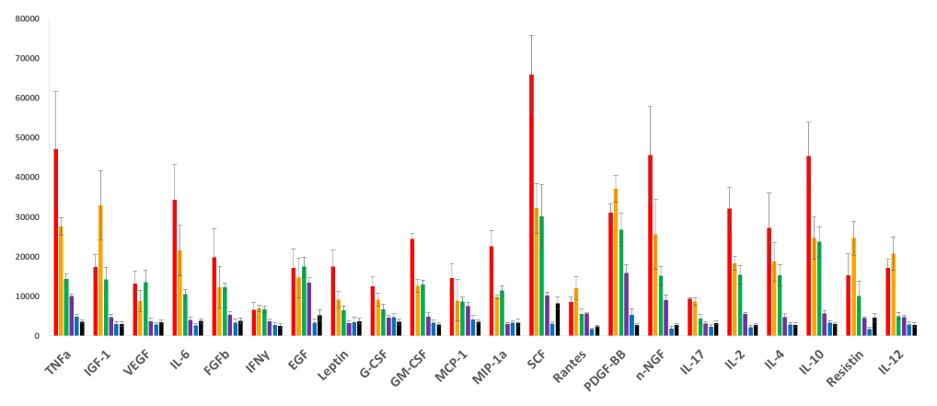


Luminescence Units

Fig S1 FASN inhibition prevents pro-inflammatory cytokine increases in a diet induced model of NASH

Cytokine/chemokine levels measured from serum collected at the end of the study (57 days). Mean +/- SD is shown for animals on a form of Western diet containing high fat, fructose and cholesterol diet,treated with vehicle (red) or TVB-3664 at the indicated dose levels (0.3, 1, 3, 10 mg/kg in orange, green, purple, blue respectively) or on a normal diet treated with vehicle only (black).

Fig S2


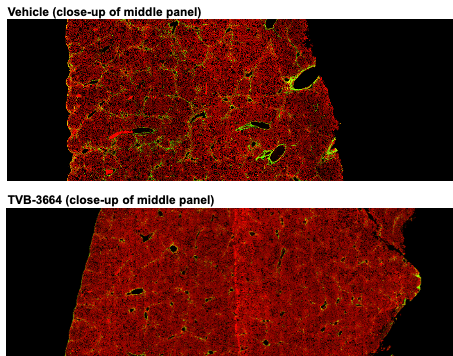


Fig S2 SHG images of representative livers from the CCl4 FAT-NASH model

Close up view of middle panel images from Fig 4. Collagen is represented in green.**Table S1: Grade Severity of Histologic Changes of Liver Sections Stained with**

**Trichrome, Adipophilin or ORO Stains in a preventative diet induced NASH model**


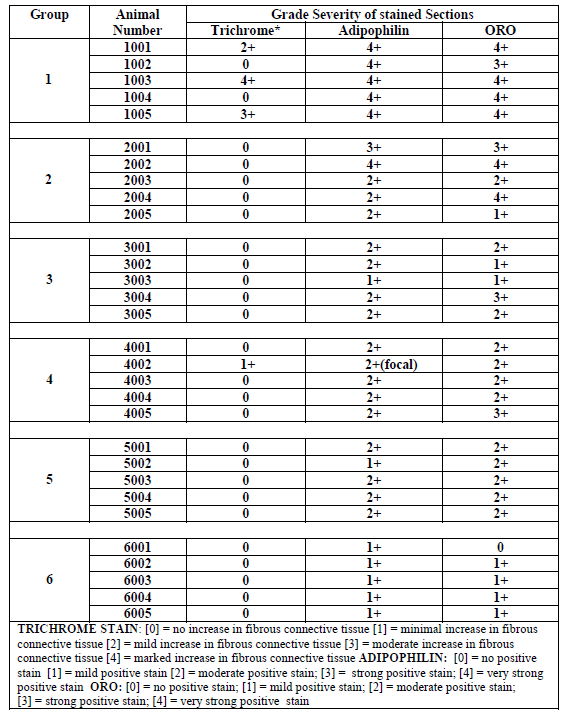


**Table S2: Histopathologic Evaluation of H&E Stained Sections of Liver in a preventative diet induced NASH model**

**
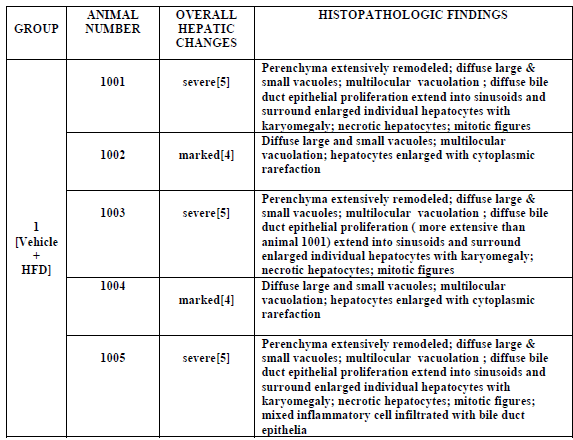
**

**Table S2 continued-1**
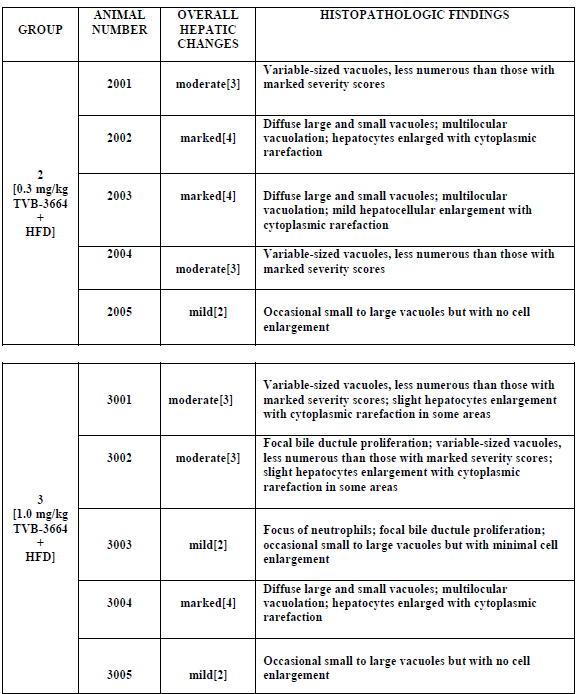


**Table S2 continued-2**
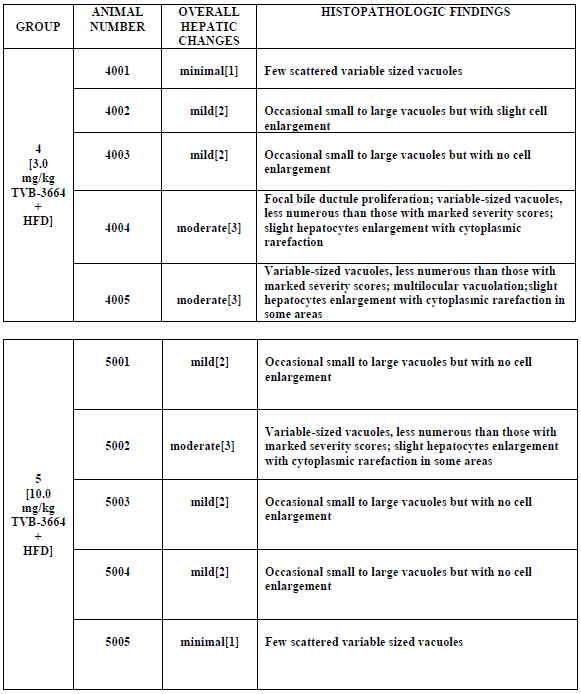


**Fig S3**

**
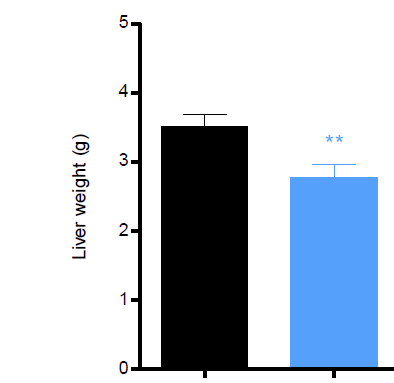

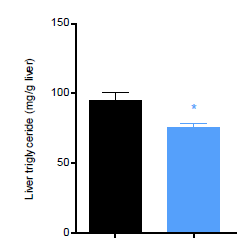
**

**
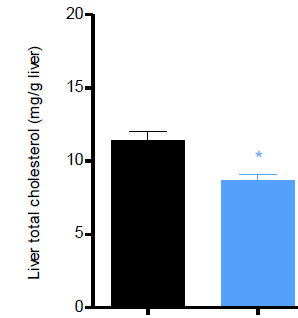
** Vehicle FASNi Vehicle FASNi

Vehicle FASNi

Fig S3 FASN inhibition reduced liver weight, liver triglycerides and liver cholesterol in a NASH diet induced therapeutic mouse model

Liver weight (upper left) and liver triglycerides normalized for liver weight (upper right), and liver cholesterol (lower left) at termination (day 56). Mean +/- SEM is shown (n=11-12/group). *p<0.05, **p<0.01, vs. Vehicle; One-Way ANOVA with Dunnet’s multiple comparison test.

**Fig S4**


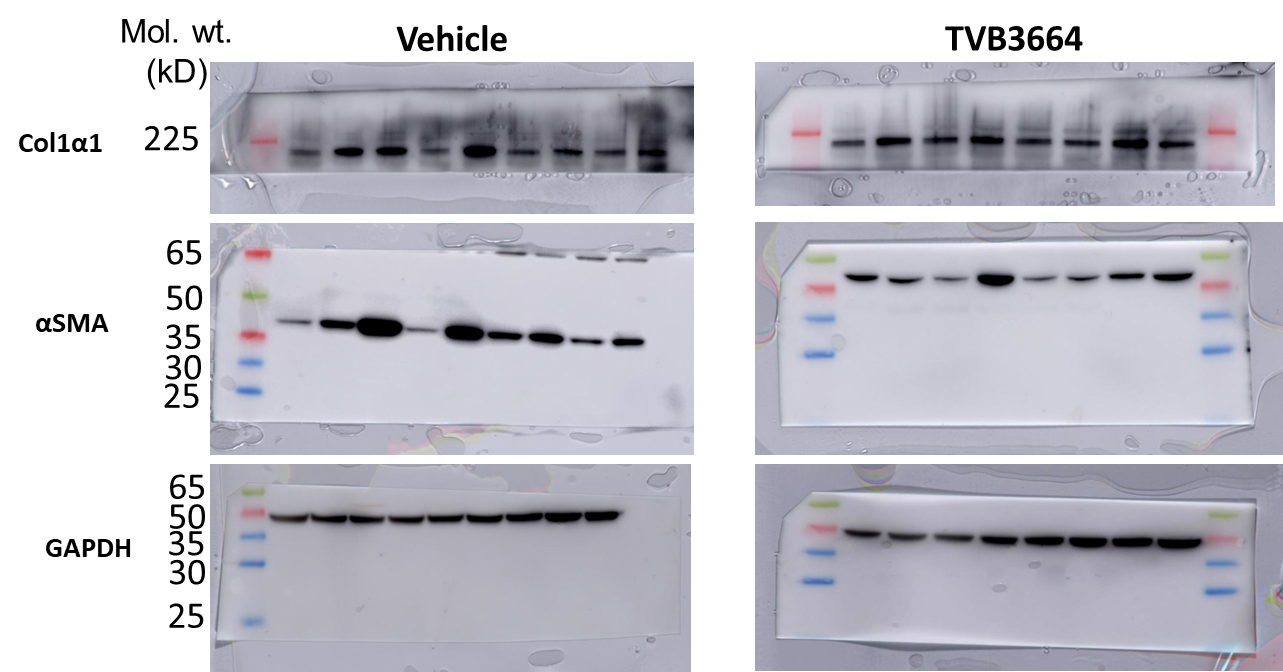


**Fig S4 legend: Key fibrogenic protein expression of TVB-3664 treated FAT-NASH mice.**

Full length Western blot (whole liver) images of either vehicle or TVB-3664 treated mice shown in Figure 5B.

The procedures are compliant with Nature digital image and integrity policies: Two different gels were run, one for vehicle and other for TVB-3664, to accommodate all samples including the protein ladder. All stages of the experiment were run in parallel including gels, Western blot procedures and image analysis, and a similar exposure time was used for membrane imaging.
